# Can we Predict the Outcomes of Arthroscopic Partial Meniscectomy?

**DOI:** 10.1007/s12178-026-10014-1

**Published:** 2026-03-30

**Authors:** Pietro Conte, Giuseppe Anzillotti, Imrane Gazali, Marco Minelli, Daniele D’Arrigo, Elizaveta Kon, Peter Verdonk

**Affiliations:** 1 IRCCS Humanitas Research Hospital, Via Manzoni 56, 20089 Rozzano, Milan, Italy; 2 Department of Biomedical Sciences, Humanitas University, Via Rita Levi Montalcini 4, 20072 Pieve Emanuele, Milan, Italy; 3 ORTHOCA, Antwerp, Belgium; 4 ASTARC Department, Antwerp University, Antwerp, Belgium; 5 MoRE Institute, Antwerp, Belgium; 6 Department of Orthopaedic Surgery, Aspetar Hospital, Doha, Qatar; 7 Department of Orthopaedic Surgery, Antwerp University Hospital (UZA), Antwerp, Belgium

**Keywords:** Meniscus, Knee, Predictors, Profiling, Morphotype, Sport medicine, Osteoarthritis, Arthroscopy, Meniscectomy

## Abstract

**Purpose of Review:**

This manuscript comprehensively reviews the most recent studies on preoperative and intraoperative factors that positively or negatively influence the clinical and radiological outcomes of arthroscopic partial meniscectomy (APM).

**Recent Findings:**

Initial research focused on baseline demographic (e.g., age, sex, BMI) and meniscal tear characteristics to predict short- and medium-term outcomes. Recent investigations, however, have broadened this scope to include previously overlooked ancillary elements, such as socio-economic and psychological factors. Furthermore, non-meniscal anatomical features—including bony alignments and morphology, and joint stability—have been studied, along with surgical-related elements like the volume of resected tissue and the impact of specific surgical techniques. Crucially, long-term studies, some with over 20 years of follow up, are now available, offering a clearer understanding of the rates of symptomatic and asymptomatic osteoarthritic progression based on different baseline characteristics.

**Summary:**

While precisely predicting the clinical and radiological outcomes of APM remains challenging, this manuscript provides a comprehensive review of the most current evidence. It aims to help surgeons more adequately treat and counsel patients presenting with meniscal tears. Future research should prioritize developing and validating predictive algorithms that integrate the multitude of success and failure factors discussed in this review.

## Introduction

Arthroscopic partial meniscectomy (APM) is one of the most frequently performed orthopedics procedures and generally provides good short-term clinical results. Nonetheless, it is associated with a higher long-term risk of osteoarthritis (OA) progression compared to meniscal repair and non-operative treatment [1, 2]. Consequently, the modern orthopedic approach adheres to the “Save the Meniscus” principle, emphasizing meniscal preservation (intended both as non-operative management and meniscal repair procedures when surgery is needed) and limiting APM to selective cases [3]. According to the 2019 ESSKA consensus, when dealing with acute traumatic meniscal tears, APM is indicated only when repair or observation are not viable options, such as for complex, highly degenerated, flap, non-reducible bucket handle, or avascular zone tears [4]. For degenerative meniscal tears, APM must not be considered as a first-line treatment: the 2016 consensus states that APM is indicated only after three months of failed conservative measures (or even earlier if a patient experiences considerable mechanical symptoms, such as locking or catching) exclusively in patients with normal X-rays (no evidence of advanced OA) and an abnormal magnetic resonance imaging (MRI) [5].

Nonetheless, APM still holds a relevant role in the orthopedic practice, with its clinical and radiological outcomes strongly depending on precisely choosing those patients with high preoperative probability of having a positive clinical outcomes and limited risk of osteoarthritic degeneration. As a result, research from the last 30 years focused on identifying those preoperative predictors such as patient-related factors (e.g. age, body-mass index (BMI), sex, symptom duration.), anatomical factors (e.g. tear configuration, concomitant lesions, morphotype.) and surgical factors (e.g. amount of resected tissue, rim integrity.) [6, 7].

**Fig. 1:**
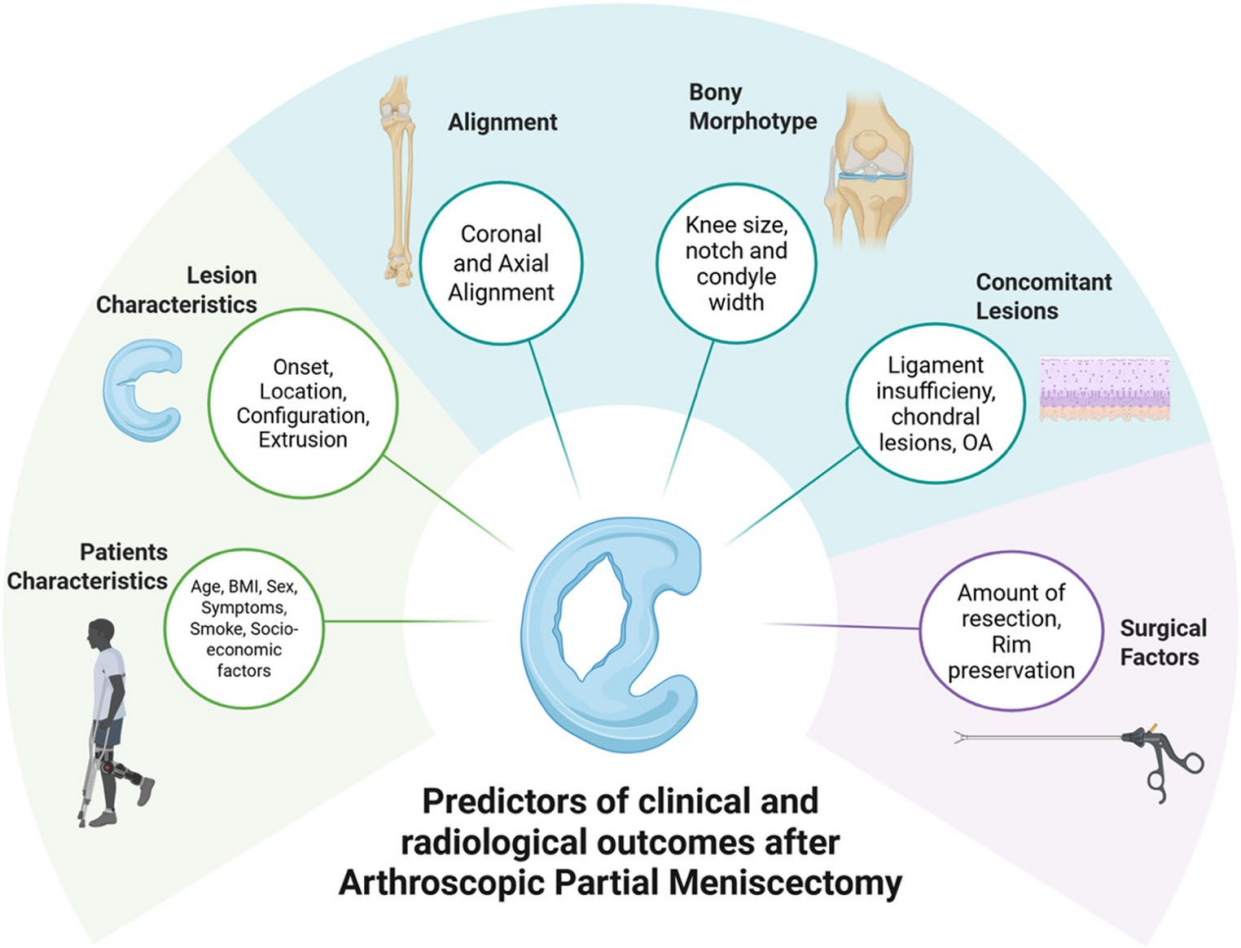
Factors influencing the clinical and radiological outcomes of Arthroscopic Partial Meniscectomy

The purpose of this review is therefore to comprehensively collect all the available studies assessing the specific factors influencing the clinical and radiological outcomes of APM with the final aim of guiding surgeons towards more precise surgical counselling when dealing with patients presenting meniscal tears (Fig. 1 and Table 1).

**Table 1:** Proposed predictors of clinical and radiological outcomes following arthroscopic partial meniscectomy

Category	Specific factor	Influence on outcome
Patient Characteristics	Older Age	Generally associated with worse post-operative clinical and radiological outcomes. No agreed cut-off available
	Obesity	Predictor of worse clinical outcomes, lower satisfaction, and higher risk of OA progression. No agreed BMI cut-off available
	Sex	No definite differences in clinical nor radiological results
	Preoperative Symptoms	Better preoperative status seems to predict positive outcomes and higher odds of reaching PASS but lower odds of reaching MCID and SCB
	Preoperative Pain	Higher baseline pain correlates with smaller clinical improvements
	Symptom Duration	Chronic symptoms are associated with smaller improvements
	Smoking	Negatively impacts PROMs for pain and function
	Psychological Factors	Anxiety, depression, and poor mental health scores predict smaller clinical improvements
	Socio-economic Factors	Lower education levels and area deprivation are significant predictors of inferior outcomes
Lesion Characteristics	Nature of Tear	Treatment of traumatic tears leads to better outcomes compared to degenerative tears
	Location	Higher rates of radiological progression after lateral APM but clinical outcomes are similar to medial procedures
	Tear Configuration	Treatment of complex tears leads to inferior outcomes; radial and flap tears may predict positive results
	Meniscal Extrusion	Higher preoperative extrusion is linked to unsatisfactory clinical outcomes. No agreed cut-off available
Bony Anatomical Factors	Coronal Alignment	Preoperative varus alignment (especially > 5.5°) seems to be a risk factor for clinical failure
	Bony Morphotype	Smaller knee size in the mediolateral axis, smaller medial femoral condyles and wider notch seem to increase risk of persistent pain after APM
Concomitant Lesions	Ligament Insufficiency	ACL-deficient knees show higher rates of radiographic degeneration and lower outcome scores after APM
	Chondral Damage	Baseline cartilage damage is a strong predictor of post-APM OA development
	Preoperative OA	Higher KL grades significantly reduce the likelihood of meaningful improvement
Surgical Factors	Amount of Resection	A greater volume of removed tissue correlates with higher rates of degenerative progression
	Rim Integrity	Preservation of a stable, adequately thick peripheral rim is critical to avoid clinical and radiological failure

*OA *osteoarthritis;* BMI *body-mass index;* PASS *patient-acceptable symptoms state;* SCB *substantial clinical benefit;* MCID *minimal clinical important difference;* PROM *patient reported outcome measures; *APM *arthroscopic partial meniscectomy;* KL *Kellgren-Lawrence

## Factors Influencing the Clinical and Radiological Outcomes of Arthroscopic Partial Meniscectomy

### Patients’ Characteristics

Several patients’ baseline characteristics have been investigated as possible factors influencing clinical and radiological outcomes of APM. Among others, the most frequently assessed are sex, age, BMI, preoperative activity levels, preoperative symptoms, smoke habit, psychological and socioeconomical factors.

Sex should not be considered as a relevant prognostic factor: the two available systematic reviews on the topic from 2010 to 2018 reported that the majorities of available studies found no difference in both clinical and radiological outcomes after APM, only few studies found worse outcomes in females while no study found worse results in males [6, 7]. Accordingly, more recent studies variably found no impact of patients’ sex [8, 9], worse clinical results in females when BMI was > 27 kg/m^2^, and lower satisfaction rates in female patients [10].

On the other hand, it is generally agreed that older age is associated with worse post-operative outcomes even if specific cutoffs have not been reported [6, 7]. Of notice, in contrast with the majority of the most recent available studies reporting older age to be a predictor of worse clinical and radiological outcomes [8, 11, 12], Tayfur et al. were amore the few finding similar clinical responses when comparing patients above and below 40 years of age [13] and Dwyer et al. reported that age was not a predictor of the probability of achieving the patient acceptable symptomatic state (PASS) for all the assessed clinical outcome measures [14].

Similarly, literature generally agrees on the fact that obesity has to be considered a predictor of worse clinical outcomes, lower satisfaction rates and higher risk of OA progression after APM [8, 10, 15–19]. Nonetheless, a 2021 systematic review and meta-analysis from Zhang et al. collected 16 studies reporting the outcomes of APM in overweight and obese patients and found that, although obesity may be associated with worse pre- and postoperative scores, there is no significative difference in scores improvements between obese and nonobese patients [20]. Furthermore, higher rates of OA development and general complications were found in overweight and obese patients but none of those differences reached statistical significance [20]. Of notice, obesity is generally measured through BMI, but this method has been criticized because it is an indirect surrogate measurement that does not always reflect true body composition, or adiposity. As a result, Dai et al., demonstrated that the adipose-to-muscle area ratio at the knee is a stronger predictor of clinical outcomes after APM when compared to BMI calculation [21].

As opposed to obesity and sedentary lifestyle, a higher baseline activity level and sport participation could be though to be correlated with better outcomes after surgery. Nonetheless, the available systematic reviews on the topic reported that none of the included studies found a correlation between sport level and clinical outcomes [7] while some studies reported higher incidence of radiographic joint degeneration after APM in patients reporting a high sport participation before surgery [6, 22, 23].

The intensity and duration of preoperative symptoms have also been assessed as potential predictors of different outcomes. Specifically, higher preoperative reported pain seems to be correlated with worse clinical improvements after APM [17, 18], specifically when present at night [24] and associated with pain sensitivity as assessed by specific questionnaires [25]. Interestingly, Gowd et al. demonstrated that higher cumulative scores at baseline (mixing specific subscales such as pain, symptoms, quality of life and indicating a general better patients’ status), despite being associated with reduced odds of achieving minimal clinical important differences (MCID) and substantial clinical benefit (SCB), correlates with greater odds of achieving patient-acceptable symptom state (PASS) [26] and the same was confirmed by Dwyer et al. [14]. Similarly, the recent METRO study, comparing 91 patients undergoing APM to 91 conservatively treated patients, reported that the only predictors of significantly better outcomes were better Western Ontario Meniscal Evaluation Tool (WOMET) scores at baseline and the fact of being surgically treated [9] confirming the pre-existing evidence from Sgroi et al. that found poor baseline WOMET scores as predictors of clinical failure after APM [27].

As for symptoms’ duration, recent studies generally agree on the fact that longer chronic symptoms are associated with smaller clinical improvements after surgery [7, 18, 28, 29] while early APM for symptomatic traumatic tears leads more positive clinical outcomes [30]. Regarding specific symptoms features, locking and catching symptoms have been historically considered predictors of positive outcomes of APM and, as a result, an indication for surgery even for degenerative meniscal tears [5, 31]. Nonetheless, the METRO study recently found no association between mechanical symptoms and post-treatment clinical outcomes posing doubts on this specific assumption [9].

A potentially modifiable factor that hinders the clinical outcomes of APM is smoking [8, 29, 32, 33]. Recently, Kraus et al. reported the outcomes of a cohort of 509 patients undergoing APM comparing smokers and non-smokers at the time of surgery and found statistically significant lower PROMs for pain and function for all time points in smokers [34]. Interestingly, smokers had also statistically significant worse mental health scores at baseline, as assessed by the Veterans Rand 12-Item Health Survey Mental Composite Score (VR-12 MCS) questionnaire, and that could be seen as an aggravating factor.

Indeed, recent research has focused on the impact of both psychological and socio-economic factors on the outcomes of different surgical procedures. Hirpara et al. analyzed more than 50.000 patients treated with APM from the TriNetX database and found that those with an anxiety or depressive disorders had higher rates of health care utilization, medical complications, opioid prescriptions, and subsequent knee surgery (revision meniscal surgery or total knee replacement) after APM [35]. Accordingly, a poor mental health condition, represented by lower VR-12 MCS and Short Form Health Survey 36 Mental Health Score (SF-36 MHS), was found as a predictors of smaller clinical improvements after APM in several recent studies [8, 11, 29].

Similarly, socio-economic factors are relevant when trying to predict the clinical outcomes of similar procedures. Indeed, lower level of education [8], lower workers’ compensation [26] and higher Area Deprivation Index values [17] have all been found to be significant predictors of inferior clinical outcomes after APM.

### Lesion Characteristics

Having addressed all the aforementioned patients’ baseline characteristics, focus should then shift to specific tears’ features that are linked to better or worse clinical outcomes after APM: tear configuration, location (medial vs. lateral), nature (traumatic vs. degenerative) and the presence or absence of meniscal extrusion are all factor that may help predict if a specific patient will benefit more or less from resection surgery.

Several classifications and terminologies can be used to describe meniscal tears (e.g. horizontal, radial, root tears, longitudinal, RAMP.) and this lack of consistency limits the possibility of assessing the different outcomes of APM for each specific tear configuration throughout the literature, thus resulting in inconclusive evidence. Indeed, in their systematic review on the topic, Eijgenraam et al. reported that 8 out 9 studies assessing the association between the type of meniscal tear and clinical outcomes found no association and that none of those studies reported the classification used to describe the included tears [7]. More recently, two studies compared the outcomes of APM performed for meniscal tears divided in root and non-root tears and found no differences in radiological and clinical outcomes [36, 37] while the OME study reported decreased odds of a clinically important improvement in pain or function for meniscal root tears [8]. When further diving non-root tears treated with APM, radial mid-body medial meniscus tears and flap tears were reported to predict positive outcomes [38] while complex meniscal tears were prognostic factors for clinically-relevant inferior outcomes [39].

The location of the tear (medial vs lateral compartment) adds complexity in classifying meniscal lesions and identifying specific risk of unsuccessful outcomes. Given the different biomechanics of the two compartments, the same tear may result in different outcomes if found and treated in the medial or lateral compartment. Indeed, it is generally thought that APM performed for lateral tears is associated with worse outcomes: long term studies from the early 2000’ found, 10 years after APM, clinical results to be similar but radiological results to be worse for lateral meniscus tears with rates of radiological changes as high as 40% [40]. Nonetheless, evidence on the topic is still contrasting with some recent studies supporting the inferior outcomes of the lateral compartment [27], others even finding worse results in procedures performed on the medial meniscus [16, 26, 41] and some reporting no differences between the two compartments [9].

Regardless of the configuration and locations of the meniscal lesion, most of the available literature supports the fact that better results can be expected when APM is performed for traumatic tears when compared to degenerative ones. Englund et al. were the first to report inferior clinical outcomes [42] and higher rates of post-operative OA [43] for degenerative tears with a risk of osteoarthritic progression found to be twice the one of traumatic tears [23]. Those results were confirmed by a recent systematic review including 32 studies and concluding that 10-years outcomes of APM are better for traumatic tears when compared to degenerative ones considering both clinical improvements and radiological OA progression [44].

To further support this concept, meniscal extrusion (considered a sign of meniscal degeneration and associated with the development of osteoarthritis in the long term [45]) is also seen as a factor negatively influencing the outcomes of APM: a study from Novaretti et al. recently described that patients with preoperative meniscal extrusion of 2.2 mm or greater had unsatisfactory clinical outcomes and progression of OA after medial APM at a minimum of 5 years follow-up [46]. Accordingly, consistence evidence supports a direct correlation between the entity of medial meniscus extrusion and the worsening of the clinical results with cut-off proposed to be of 2.05 mm [47] or 11% [39] based on the different measurements.

### Bony Anatomical Factors

#### Coronal and Sagittal Alignment

Lower-limb coronal malalignment is a key mechanical determinant of compartmental load and is associated with knee OA incidence and progression in longitudinal cohorts [48, 49]. Because APM removes load-sharing meniscal tissue, the interaction between meniscal deficiency and coronal alignment is biomechanically relevant [50, 51]. In cadaveric testing, varus alignment increased medial compartment contact pressure and influenced contact area after sequential medial meniscus resection compared with neutral/valgus alignment [52].

Clinically, a cohort study on degenerative medial meniscal tears reported that greater preoperative varus alignment was associated with significantly worse post-operative outcomes at a mean of 32 months after APM [47]. Similarly, in a mid- to long-term clinical series of APM for medial meniscal tears (mean 9.8 years of follow up), a lower HKA was found to be a significant risk factor of clinical failure with a cutoff value reported to be of 5.5° varus [12].

Nevertheless, coronal alignment may also change as a result of meniscectomy, as a clinical study reported increased varus alignment after medial meniscectomy, with the magnitude related to the extent of meniscal resection [53].

Hence, supported by the aforementioned evidence, bony procedures raised in order to reduce the amount of deformity. High tibial osteotomy (HTO) is a well-established strategy to shift the mechanical axis laterally and unload the medial compartment in varus knees with medial compartment disease. Interestingly, in patients undergoing medial opening-wedge HTO, a history of APM did not adversely affect midterm to long-term outcomes in a retrospective study [54]. As a result, for varus knees in which degenerative meniscal pathology coexists with overload features such as extrusion, contemporary clinical literature proposes realignment (when indicated) as part of a load-modifying strategy rather than relying on isolated meniscal resection [47].

As for sagittal alignment, a recent systematic review and meta-analysis found that increased posterior tibial slope (PTS) and “meniscal slope” parameters are associated with higher risk of developing specific meniscal injuries, specifically lateral meniscal tears in acute context and medial meniscus tears and posteromedial root tears in the degenerative context [55]. At present, the impact of PTS on the outcomes of APM has never been assessed and reported while most of the literature focuses on the impact on anterior cruciate ligament (ACL) tears and ACL reconstruction failure. Nonetheless, it is reasonable to think that a higher PTS, being associated with altered knee biomechanics and a resulting increased rate of meniscal tears, could represent a predictor of worse clinical outcomes after APM but such assumption will have to be confirmed by future studies on this specific topic.

#### Bony Morphotype

Beyond simple axis measures, three-dimensional (3D) tibiofemoral bone morphology has been investigated as a determinant of symptoms after meniscal tissue loss and therefore a possible predictor of different outcomes after APM.

MRI-based bony morphology has also been linked to tear complexity, as complex medial meniscus tears were associated with a biconcave medial tibial plateau configuration [56].

Grammens et al. were the first to investigate the relationship between specific knee morphotypes and intra-articular pathology, reporting that a “small medial femoral condyle” morphotype seems to be associated with medial compartment degeneration and distinct morphological characteristics compared with controls [57]. The same group later performed a study comparing patients with positive outcomes after APM (KOOS pain > 75 points) and those with recurrent pain (KOOS pain < 75 points) thus diagnosed with a post-meniscectomy syndrome: the study found that patients with persisting pain had significantly smaller knees in the mediolateral axis, a wider femoral notch and a smaller medial femoral condyle compared with those performing well after APM [58]. Furthermore, the authors proposed a morphology-based predictive algorithm that could predict medial post-meniscectomy syndrome (MPMS) in neutrally aligned patients: the authors reported that a diagnostic sensitivity of 74.9% and a specificity of 81.0% could be reached just by combining the patients’ tibiofemoral morphology to just four anamnestic parameters (gender, age, weight and height) [58]. Interestingly, MPMS risk was not driven by BMI but rather from the ratio between higher body mass over a smaller knee.

These findings support the broader concept that intrinsic bony geometry and shape may influence compartmental mechanics and the clinical phenotype after meniscal tissue resection, complementing alignment- and extrusion-based risk stratification. Given the demonstrated associations between plateau shape, tibial slope, and meniscal tear patterns, morphology-informed assessment may be particularly relevant in selecting or counseling patients in whom meniscal tissue loss could interact unfavorably with underlying bone geometry [55, 56, 58, 59].

### Concomitant Lesions

#### Ligament Insufficiency

Meniscal pathology requiring APM frequently coexists with ligamentous insufficiency, particularly involving the anterior cruciate ligament (ACL). An increase in anterior tibial translation and a decrease in rotational stability has been observed when ≥ 46% of the posterior horn of the medial meniscus (PHMM) has been removed [60]. The biomechanical role of the meniscus as a secondary stabilizer becomes even more relevant in the ACL-deficient knee [61].

A systematic review suggested that meniscal deficiency is associated with an increased risk of degenerative changes, regardless of ACL reconstruction (ACLR) status [6]. However, even higher rates of radiographic degenerative changes and lower patient-reported-outcome-scores were observed in ACL-deficient knees compared to ACL-intact knees after meniscectomy [6]. Similarly, Sgori et al. found that the presence of untreated concomitant ACL pathology was independently correlated with worse postoperative patient-reported knee function after APM [27] and Sofu et al. concluded that the presence of ACL either partially ruptured or degenerated with increased laxity should be considered a negative clinical predictor after APM specifically performed for acute medial meniscal tear in patients older than 60 years of age [15].

As for coronal plane instability, evidence that specifically evaluates clinical outcomes after APM in the presence of collateral ligament insufficiency is scarce in the literature. Figueroa et al. described the incidence and treatment patterns of meniscal tears in multi-ligament knee injuries (MLKI) rather than prognosticating outcomes of APM as a standalone procedure in unstable knees: in a cohort of MLKI reconstructions, meniscal lesions were common (67,1%), with a predominance of lateral meniscus tears, particularly in ACL + medial-side injury patterns [62]. This paucity of data does not allow to draw conclusions on whether isolated collateral ligament insufficiency independently drives worse outcomes after APM.

#### Cartilage Lesions and Osteoarthritis

Cartilage lesions are frequently encountered in knees undergoing APM; however, the impact of those on the clinical outcomes and the best management of concomitant chondral pathology in this setting remains insufficiently studied. It is generally agreed that pre-existing chondral damage is a predictor of post-APM OA development and the majority of the studies included in the systematic review from Salata et al. supported this assumption [6]. Similarly, six out of the ten studies included in the systematic review from Eijgenraam et al. concluded that the presence of chondral damage predicted worse clinical outcomes after APM in a manner that may be driven by age since all the studies assessing patients over 45 years of age found this negative association [7]. More recent evidence supports the fact that baseline chondral damage is a predictor for both worse clinical outcomes and higher OA progression rates [8, 27].

On the contrary, until recently there were no high-quality studies specifically evaluating the impact of treating concomitant cartilage lesions at the time of APM. However, long-term data from the ChAMP randomized controlled trial indicate that the debridement of unstable chondral lesions encountered during APM does not result in improved patient-reported outcomes, reduced pain, lower rates of subsequent surgery, or less radiographic progression of osteoarthritis at nine years compared with observation alone [63].

Additionally, to date no studies have specifically evaluated the impact of cartilage repair procedures, such as microfracture or restorative cartilage techniques, performed concomitantly with APM on clinical or structural outcomes.

On the other hand, preoperative OA has been consistently identified as an important predictor of worse clinical outcomes after APM. The systematic review from Eijgenraam et al. reported that patients with more advanced radiographic OA experienced inferior postoperative outcomes compared with those without OA, although the overall level of evidence was moderate [7]. Recent cohort studies further support this association: increasing Kellgren–Lawrence (KL) grade has been shown to reduce the likelihood of achieving clinically meaningful improvement after APM [28]. Similarly, Hong et al. reported that preoperative KL grade ≥ 2 is a risk factor for clinical failure at mid- to long-term follow-up after APM for medial meniscal tears [12].

Compartment-specific degeneration also appears to influence outcomes. High-grade medial compartment OA (KL grades 3–4) has been associated with poorer patient-reported outcomes, whereas the absence of patellofemoral OA may have a protective effect when performing medial APM [29]. Accordingly, in older patients undergoing APM, degenerative changes of the patellofemoral joint have been found to predict persistent pain and limited functional improvements [15].

In middle-aged patients presenting with degenerative meniscal symptoms, long-term randomized controlled trials have shown that APM does not confer a protective effect against the development of radiographic or symptomatic osteoarthritis when compared with non-operative treatment: at 10-year follow-up, the prevalence of radiographic osteoarthritis and patient-reported outcomes were comparable between patients treated with APM and those treated with structured exercise therapy alone [64].

### Surgical-Related Factors

#### Amount of Resection

The extent of meniscal tissue removed during APM is one of the most consistently reported surgical factors associated with structural joint changes. Imaging-based studies using semi-quantitative MRI assessment have demonstrated that a greater amount of meniscal resection correlates with a higher burden of degenerative joint disease [65]. In the Osteoarthritis Initiative cohort, higher meniscus resection scores were associated with worse knee abnormalities on follow-up MRI [65]. Earlier clinical and radiographic studies have consistently emphasized that more conservative meniscectomy techniques are associated with more favorable long-term joint preservation compared with subtotal or total meniscectomy [6, 7].

On the other hand, preservation of a stable peripheral meniscal remnant is commonly advocated during APM, as excessive removal of meniscal tissue has been associated with greater structural joint abnormalities on MRI [7].

#### Type of Resection

Evidence specifically comparing clinical outcomes according to tear morphology or resection pattern (e.g. inferior flap, superior flap) after APM remains limited with most outcome studies focusing on the presence of meniscal resection rather than detailed resection configuration. Of notice, in 2016 Brown et al. performed a cadaveric study and found that resection of a single inferior leaflet after a horizontal medial meniscal tear preserved much of the original biomechanical function of the meniscus while resection of both leaflets lead to a significant increase in contact pressure dispersed over the same contact area, which resulted in an undesirable biomechanical environment [66]. Nonetheless, literature on the topic is scarce and no high-quality comparative data are available to support the superiority of one flap resection over another with respect to clinical or structural outcomes after APM.

On the other hand, preservation of a stable peripheral meniscal remnant is commonly advocated during APM, as excessive removal of meniscal tissue has been associated with greater structural joint abnormalities on MRI [7].

Clinical literature further emphasizes that the goal of APM should be removal of unstable fragments while maintaining a smooth, stable, and adequately thick rim. However, despite the widespread acceptance of rim preservation as a surgical principle, high-quality studies directly comparing outcomes based on rim quality or thickness after APM are lacking. As such, recommendations regarding rim preservation are primarily supported by biomechanical rationale and indirect clinical evidence rather than by dedicated comparative outcome studies.

## Personal Observations and Clinical Insights

### Authors Experience, Current Practice and Opinions

The authors contend that patient profiling is the most effective strategy for optimizing outcomes in APM. Success hinges on accurately differentiating the nature of the lesion (degenerative vs. traumatic) and understanding the clinical context, including bony morphology, patients’ demographics, and expectations.

In the authors’ opinion, degenerative tears typically result from a combination of joint overload (driven by malalignment, high activity levels, or an elevated body mass over a small knee [58]) and genetic predisposition. Since genetic factors remain under-researched, future studies should investigate specific risk factors and comorbid pathologies, such as lumbar disc herniation, which the authors frequently observe alongside degenerative tears in younger patients.

Once a degenerative tear is diagnosed, conservative management is mandatory for three to six months and should include physical therapy, orthotic insoles, and injections (corticosteroids, polynucleotides, or orthobiologics [67, 68]). APM should be reserved for patients who fail conservative treatment, demonstrate a high probability of clinical improvement and present a low risk of accelerated osteoarthritic progression, considering all the different factors discussed in this review and reported in Table 1. With such patients, specialists must provide thorough counseling and education before proceeding.

Similarly, lateral degenerative tears require extreme caution; these often develop in the meniscal body or anterior horn due to repetitive hyperextension and are frequently associated with parameniscal cysts. In these cases, the authors prefer an all-outside suture of the meniscal body to seal the cyst.

As for traumatic tears, repair should be performed whenever feasible. However, clinicians must acknowledge high failure rates, particularly in red-white and white-white zone tears and within the athletic population. Athletes should be counseled that a repaired meniscus lacks the structural integrity and resistance of native tissue, making failure during a return to sport a distinct possibility [69]. Conversely, early APM may be considered for small radial tears in the lateral meniscal body of athletes; a limited resection can create a stable, tapered surface that reduces the risk of further crack propagation.

In conclusion surgeons must recognize that, when APM is performed correctly on the appropriate candidate, the procedure itself is not the primary driver of osteoarthritis. Rather, the long-term prognosis is dictated by the initial meniscal lesion and the underlying predisposing factors that should therefore be correctly identified and addressed.

### Future Directions and Unsolved Issues

The authors advocate a novel holistic approach to the both the meniscus and the whole scientific evidence regarding its treatment. The meniscus must not be seen as “thing” to be “fixed,” but as a part of a living system whose symptoms emerge from tissue biology, mechanics, behavior, and expectations interacting over time. Rather than pursuing one more variable that “predicts outcome,” we should aim to understand trajectories of who improves, who doesn’t and why, acknowledging that structure and pain often speak different languages. Methodologically, this means standardized biological and morphological phenotyping [70], long follow-up, and externally validated models that are interpretable enough to support a conversation in clinic, not just a p-value in a chart. Conceptually, it means reframing success from procedure performed without complication to function regained, and from imaging normalized to quality of life restored. Ultimately, progress will come not from sharper arguments for or against a technique, but from a more precise guideline: matching the right intervention, to the right person, at the right time.

### Current Limitations

The findings of this review should be interpreted in light of several inherent limitations. First, the reporting of anatomical factors such as meniscal extrusion, BMI, alignment and morphology was notably limited and inconsistent across the included studies. A significant portion of the literature failed to strictly define anatomical parameters or utilized varying measurement techniques. This lack of standardization precluded a robust quantitative analysis of how specific anatomical characteristics correlate with clinical outcomes. Furthermore, there was substantial heterogeneity regarding outcome measures (different PROMs used in the included studies) and classification systems. The use of diverse functional scoring scales and differing criteria for complication or failure rates restricted the ability to pool data for a possible meta-analysis, necessitating a qualitative synthesis of the results. Finally, the current evidence base is dominated by retrospective data. The majority of included studies were retrospective case series, which are inherently susceptible to selection bias, information bias, and confounding variables. The paucity of prospective, randomized controlled trials limits the strength of the causal inferences that can be drawn from this research and will need to be addressed in future studies.

## Conclusion

Predicting the clinical and radiological outcomes of APM remains an elusive goal, as individual responses to tissue loss vary significantly. This uncertainty is driven by complex biomechanical and biological factors that are not yet fully understood. Nonetheless, patient profiling can bridge this gap; surgeons can optimize outcomes by meticulously assessing tear morphology, the biomechanical context of the injury, and patient-specific characteristics. This review examines these critical factors to provide a framework for maximizing the success of APM.

## Data Availability

No datasets were generated or analysed during the current study.
